# Terminal quadrifurcation of the aorta: A case report

**DOI:** 10.4102/sajr.v27i1.2564

**Published:** 2023-01-30

**Authors:** Siviwe S. Mpateni, Jacques Bence, Richard D. Pitcher, Michelle Da Silva

**Affiliations:** 1 Department of Radiodiagnosis, Faculty of Health Sciences, Stellenbosch University, Cape Town, South Africa

**Keywords:** interventional radiology, vascular anatomy, trauma, aorta, angiography

## Abstract

Congenital anatomical variations of the terminal aorta are rare. Given the increasing number of endovascular and laparoscopic procedures, such variations are likely to assume greater clinical significance. A 15-year-old male sustained a pelvic vascular injury following a stab to the left gluteus. Computed tomographic angiography and digital subtraction angiography demonstrated a left superior gluteal artery pseudoaneurysm and absence of the common iliac arteries consistent with congenital quadrifurcation of the terminal aorta. The patient was subsequently treated with endovascular coil embolisation with a good angiographic and clinical outcome.

**Contribution:**

Terminal aortic variants are rare and given the increasing number of interventional endovascular procedures performed in the aorta, an awareness of the potential anatomical configurations of the distal aortic branches is of increasing relevance. The authors describe the imaging findings of one such anatomical variant.

## Introduction

The normal distal aorta bifurcates into the left and right common iliac arteries at the lower border of the L4 vertebral body.^1^ The two common iliac arteries then course anterior and slightly to the left of the L5 vertebral body before bifurcating into the internal and external iliac arteries at the level of the pelvic inlet.^1,2^

Congenital anomalies of the iliac arteries are exceedingly rare, with a reported incidence of only six cases in angiography studies of 8000 patients.^2,3^ Terminal aortic quadrifurcation results from congenital absence of the common iliac arteries likely due to formation of an abnormal communication between the 5th lumbar intersegmental, dorsolateral, and descending umbilical arteries of the dorsal aorta.^2^ This anatomical variation is asymptomatic; however, awareness of this anomalous configuration is important in the planning of endovascular procedures, obstetric surgery and organ transplantation.^4,5,6^

## Patient presentation

**FIGURE 1:**
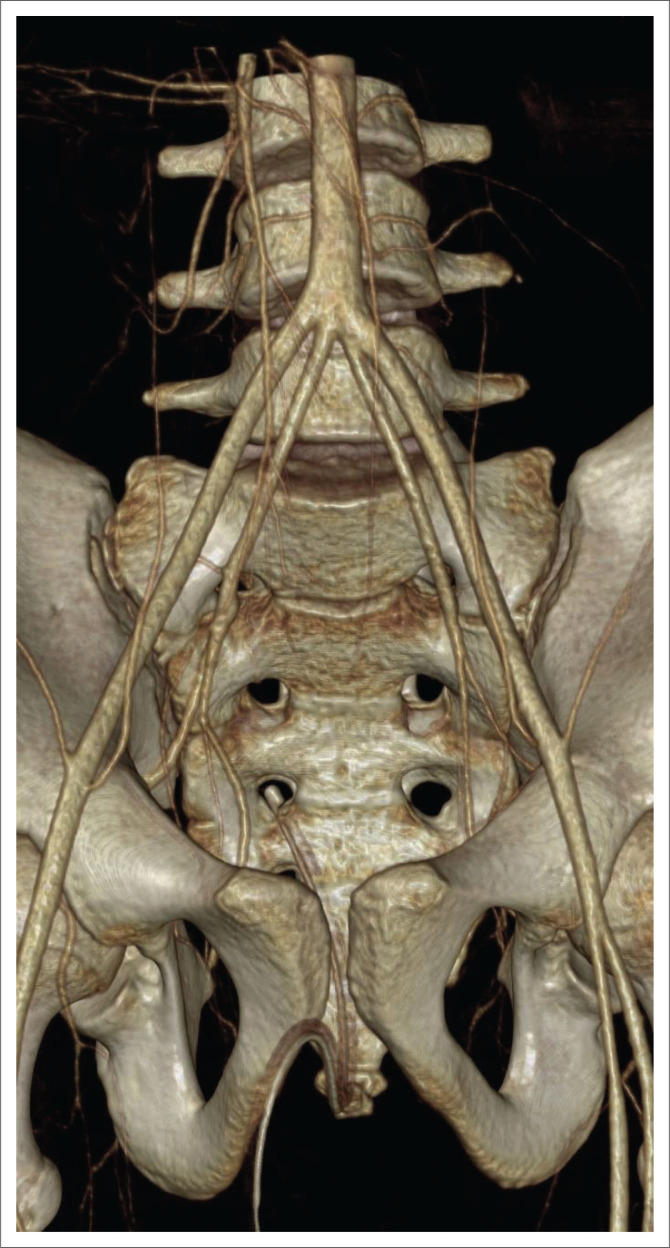
A 3D computed tomography angiogram of the pelvis depicting quadrifurcation of the terminal aorta.

A 15-year-old male was referred to the tertiary emergency centre with active haemorrhage after a left gluteal stab. Bleeding was controlled with two inflated wound-track Foley’s catheters and urgent pelvic CT angiography was performed. This demonstrated a traumatic pseudoaneurysm of the left superior gluteal artery and incidental absence of the common iliac arteries with the abdominal aorta terminating as bilateral internal and external iliac arteries at the superior margin of the L5 vertebral body (Figure 1).

**FIGURE 2:**
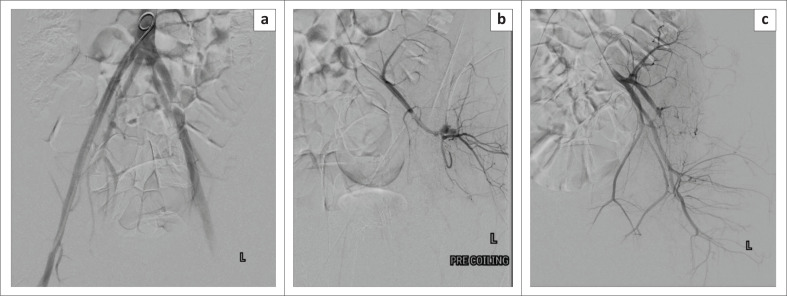
(a) Digital subtraction angiogram of the terminal aorta demonstrates bilateral absence of the common iliac arteries. (b) Digital subtraction angiogram of the posterior division of the left internal iliac artery demonstrating the traumatic pseudoaneurysm of the superior gluteal artery. (c) Digital subtraction angiogram of the left internal iliac artery demonstrated successful post-embolisation exclusion of the pseudoaneurysm.

The patient was then referred for endovascular coiling of the pseudoaneurysm by our trauma surgery team. Arterial access was obtained in the right common femoral artery under sonographic guidance. Subsequent digital subtraction angiogram of the distal abdominal aorta was performed to guide selective catheterisation of the left internal iliac artery. Super-selective catheterisation of the posterior division of the left internal iliac artery demonstrated a superior gluteal artery pseudoaneurysm, which was successfully embolised using three pushable coils (one 4 mm × 14 cm and two 4 mm × 5 cm). There was good preservation of the remaining left internal iliac branches (Figures 2 a–c).

The wound-track Foley’s catheters were deflated in the angiography suite and there was no further haemorrhage. The patient was discharged from hospital by our trauma team 2 days later.

## Discussion

The embryological development of foetal vascularisation begins in the 3rd week by a process of vasculogenesis and angiogenesis.^2^ The abdominal aorta forms by the fusion of the paired dorsal aorta in the 4th week.^1,2^ Subsequently, four paired dorsal segmental arteries originate from the abdominal aorta to form the upper lumbar arteries and the fifth lumbar artery gives rise to the bilateral common iliac arteries.^2^ At 3 months gestation definitive vascularisation is achieved.^1^

The normal distal aorta bifurcates into the left and right common iliac arteries at the lower border of the L4 vertebral body in most individuals.^1,5^ The two common iliac arteries then course anterior and slightly to the left of the L5 vertebral body before bifurcating into the internal and external iliac arteries at the level of the pelvic inlet.^1^

Variations to the configuration of the terminal aorta and iliac arteries are far rarer than the thoracic aorta and complete failure of bilateral common iliac arteries formation is extremely rare.^5,7^ The incidence of distal aortic and iliac anomalies is unknown, however, a study of 8000 patients performed by Grebe and colleagues revealed only six cases with iliofemoral anomalies.^8,9^

This rare anatomical variation is often found incidentally but carries clinical implications in several open and endovascular interventions, such as the selection of distal stent landing zone in endovascular aortic aneurysm repair,^6^ emergent surgical ligation of the internal iliac arteries to arrest intra-operative haemorrhage^5^ and selection of pelvic host vessels in renal transplantation.

Pham et al. describe a novel endovascular approach to preserve internal iliac artery flow during endovascular aortic aneurysm repair in a patient with bilateral absence of the common iliac arteries by using an iliac branched aortic graft device to repair the aneurysm. They describe selective preservation of a single internal iliac branch to perfuse the pelvis and embolisation of the contralateral internal iliac branch prior to graft deployment.^10^

With the increasing number of endovascular and laparoscopic pelvic procedures performed, such variations are likely to be encountered with greater frequency and interventionists should be prepared to tailor their procedural approach to accommodate such anomalies. This is to the best of our knowledge the first case report demonstrating the diagnostic imaging features of this anatomical variation on the African continent. Reporting of this finding when encountered is encouraged.

### Conclusion

Consideration of this rare anomaly in the planning and execution of open surgical and endovascular arterial procedures is recommended.
